# Review of computer-generated hologram algorithms for color dynamic holographic three-dimensional display

**DOI:** 10.1038/s41377-022-00916-3

**Published:** 2022-07-26

**Authors:** Dapu Pi, Juan Liu, Yongtian Wang

**Affiliations:** Beijing Engineering Research Center for Mixed Reality and Advanced Display, School of Optics and Photonics, Beijing Institute of Technology, Beijing, 100081 China

**Keywords:** Displays, Imaging and sensing

## Abstract

Holographic three-dimensional display is an important display technique because it can provide all depth information of a real or virtual scene without any special eyewear. In recent years, with the development of computer and optoelectronic technology, computer-generated holograms have attracted extensive attention and developed as the most promising method to realize holographic display. However, some bottlenecks still restrict the development of computer-generated holograms, such as heavy computation burden, low image quality, and the complicated system of color holographic display. To overcome these problems, numerous algorithms have been investigated with the aim of color dynamic holographic three-dimensional display. In this review, we will explain the essence of various computer-generated hologram algorithms and provide some insights for future research.

## Introduction

Display technology is very important for humans to acquire information. However, traditional two-dimensional (2D) display technology can only display a 2D projection image from one side of the three-dimensional (3D) scene, which loses the depth information and affects the 3D spatial information acquisition. In recent years, 3D display technology has attracted more and more attention. Among all existing 3D display technologies, holographic 3D display is regarded as the most promising 3D display technology because it can reconstruct all information of a real or virtual scene and bring no visual fatigue^1^.

**Fig. 1:**
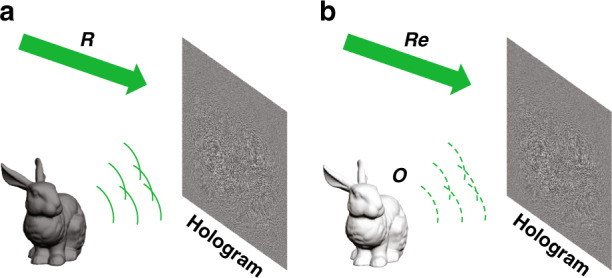
The schematic of hologram recording and reconstruction process. **a** Recording; **b** reconstruction.

Holography was invented by Denis Gabor in 1947 to improve the resolution of electron microscopy^2^. Like his name representing the meaning of whole, holography is a technique to record and reconstruct all the physical information of a 3D scene based on interference and diffraction theory as shown in Fig. 1. In recording process, the object light *O* and the reference light *R* interfere in the hologram plane and the interference fringe *I* is recorded on the hologram as shown in Eq. (1). In reconstruction process, the hologram is illuminated by the reconstruction light *Re*. If the reconstruction light *Re* is chosen as the reference light *R*, the third term of the diffraction light *U* is, up to a multiplicative constant, an exact duplication of the original object light *O* as shown in Eq. (2), which indicates that the original object can be reconstructed successfully. Due to the invention of holography, Denis Gabor won the Nobel Prize in Physics in 1971. However, the application of holography was restricted in many years because on-axis holography has inability to solve the problem of the twin image. In 1962, Leith and Upatnieks separated the twin image by increasing the carrier frequency of the reference light, thereby greatly expanding the applicability of holography^3–5^. In the same year, Denisyuk invented the reflection hologram, which can be reconstructed by white light because of the high degree of selectivity to wavelength^6,7^. Afterwards, Benton invented the two-step rainbow hologram^8^. Rainbow hologram can be reconstructed by white light because it minimizes the blurring caused by the dispersion of the transmission hologram via abandoning the parallax information in the vertical direction.

1 $$I = \left| {O + R} \right|^2 = \left| O \right|^2 \,+\, \left| R \right|^2 \,+\, OR^ \ast \,+\, O^ \ast R$$

2 $$U = \left| O \right|^2R + \left| R \right|^2R + O\left| R \right|^2 \,+\, O^ \ast R^2$$

**Fig. 2:**
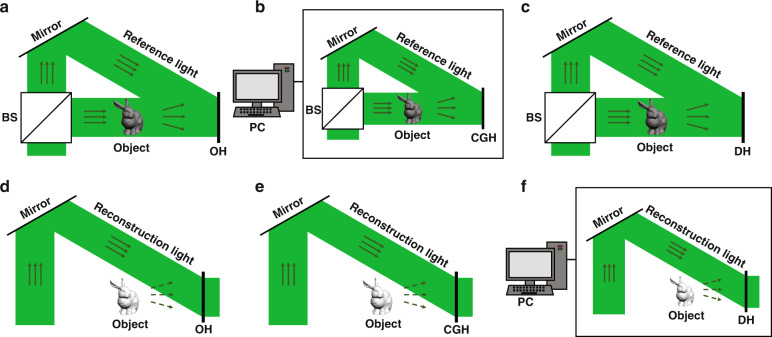
The schematic of hologram recording and reconstruction process. **a**, **d** Optical holography; **b**, **e** computer generated holography; **c**, **f** digital holography, where BS is the beam splitter and PC is the personal computer.

The traditional optical holography relies on the optical systems and photosensitive materials to complete recording and reconstruction process as shown in Fig. 2a and 2d. Optical holograms (OHs) are usually static holograms and have strict requirements on the stability of the optical systems, which restrict the application of optical holography in dynamic holographic 3D display. With the development of computer and optoelectronic technology, computer-generated holography has become an international research hotpot. In computer-generated holography, the recording process can be simulated by computers and the reconstruction can be realized by loading the computer-generated holograms (CGHs) on the spatial light modulators (SLMs) with coherent light illumination as shown in Fig. 2b and 2e. Compared with optical holography, computer-generated holography can record not only real objects but also virtual objects without complex optical systems and can realize dynamic holographic 3D display with the help of refreshable SLMs^9–13^. Because of these advantages, computer-generated holography is a future-oriented 3D display technology and can be used in many fields, such as education, entertainment, military and medical treatment^14,15^. In addition, digital holography, which records digital holograms (DHs) by sensors in optical systems and reconstructs optical wavefronts digitally as shown in Fig. 2c and 2f, has also been presented and applied in many fields, such as quantitative phase imaging and optical metrology^16,17^.

In 1960s, Kozma and Kelly combined computer and spatial frequency domain filtering technology to design matched spatial filters artificially^18^, which laid the foundation for computer-generated holography. In 1966, Lohmann and Brown devised detour phase method to encode the optical wavefronts into holograms by computer^19^. In 1967, Lohmann and Paris applied the fast Fourier transform (FFT) algorithm to calculate the Fourier transform CGH^20^, which greatly shortened the calculation time. In 1974, Lesem et al. proposed kinoform, which has high diffraction efficiency and holds a very important status in current CGH technology^21^. Since 1980s, various CGH algorithms have emerged in succession^22–28^. Meanwhile, novel modulation devices, such as acousto-optic modulators^29^, digital micromirror devices^30^, and liquid crystal displays^31^, were applied in computer-generated holography. In the last decade, with the rapid development of computer and material, computer-generated holography has made great progress and is expected to be commercialized in a near future^32^.

At present, the difficulties faced in computer-generated holography mainly focus on the following aspects: Firstly, the current calculation algorithms are not fast enough; secondly, the existing algorithms have the problem of limited reconstruction quality; thirdly, the traditional color holographic display system is complicated; finally, the unwanted terms and modulation accuracy of the SLM hinder the holographic display effect. In this paper, we provide a review of various CGH algorithms which aim to overcome the above difficulties. Organization of the paper is given as follows. Following the introduction, we review three main fast calculation algorithms in Section 2. In Section 3, we discuss various CGH optimized algorithms which aim to enhance the image quality and suppress the speckle noise. In Sections 4, we introduce some color-multiplexing coding (CMC) methods. Then we report some technologies to eliminate the unwanted terms and achieve accurate modulation in Section 5. Finally, conclusions and suggestions for future work are provided in Section 6.

## Fast calculation

**Fig. 3:**
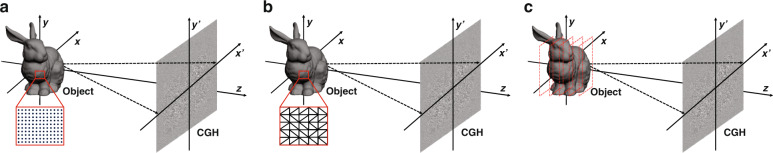
Diagram of CGH calculation method. **a** Point-based method; **b** polygon-based method; **c** layer-based method.

In CGH calculation, the 3D object is always decomposed into numerous primitives and the hologram is obtained by superposing the fringe patterns (FPs) of all primitives in the hologram plane. Therefore, the computation involved in CGH generation is huge and it is still a great challenge to realize dynamic holographic 3D display, especially when the 3D object is complicated and the size of the hologram is large. In the last decades, numerous fast computation algorithms have been proposed to accelerate CGH generation. From the perspective of the computational primitive, the algorithms can be divided into three main categories: (a) point-based method^33^, (b) polygon-based method^34^ and (c) layer-based method as shown in Fig. 3.

### Point-based method

Point-based method, where the 3D object is represented by millions of points, is a simple and widely used method to calculate the CGHs. In calculation, each object point is regarded as a self-luminous point source and emits spherical wave irradiating the hologram plane. The complex amplitude distribution in the hologram plane can be obtained by superposing the FPs of all object points. From above, it can be easily seen that the main calculation involved in point-based method is the generation of the FPs. Hence, the computation burden can be reduced dramatically if the FPs of all possible object points can be calculated in advance and stored in the computer. Inspired by this idea, Lucente proposed look-up table (LUT) method^22^, which consists of off-line computation and on-line computation. In off-line computation, the FPs of all possible object points are pre-calculated and stored in a table. In on-line computation, the CGHs are generated by reading and superposing the pre-stored FP of each object point after being multiplied by its amplitude. The LUT method speeds up the hologram generation compared with point-based method and opens a door in terms of trading memory usage for computation speed. However, the required memory usage to store the pre-calculated FPs in LUT method is too large.

In order to reduce the memory usage of LUT, novel-LUT (N-LUT) method^35^ was proposed. In N-LUT method, a 3D object is divided into multiple 2D sliced planes along the axial direction, and only the FP of the center object point in each sliced plane, called principal fringe pattern (PFP), is pre-calculated and stored in a table. The FPs of other object points can be calculated by shifting the PFP in the same sliced plane according to the relative position relation in space coordinates, and the CGHs are obtained by summing the shifted PFPs of all object points after being multiplied by the corresponding amplitude. Although the memory requirement of N-LUT method reduces dramatically compared with LUT method, it is still large and affects the speed of data acquisition seriously. For some desired 3D objects, the neighboring object points have the same intensity and depth value. Based on this phenomenon, the N-point PFPs are generated by accumulating the FPs of the neighboring object points in off-line calculation instead of on-line calculation^36^. In this way, the amount of on-line calculation is reduced in comparison with N-LUT method. Although this method is effective to accelerate CGH calculation, it is not a common method for all desired 3D objects. Therefore, scholars implemented further research to obtain a better balance among calculation speed, memory usage, and precision. The non-uniform sampling is an effective way to reduce the storage amount without extra on-line computation^37,38^. Nevertheless, the stored data for a single depth plane is still a 2D matrix.

The PFP is a function of the radius and independent of the azimuth. Based on this phenomenon, scholars proposed retrieving the PFP of a single depth plane by its radial line, which is a one-dimensional (1D) vector^39–41^. However, this method may lead the missing of some pixels in the PFP. Along this way, split-LUT (S-LUT) method^42^ was proposed, where a smart approximation is used and the FP of each object point is split by the horizontal and vertical modulation factors. In on-line computation, the FPs can be calculated by multiplying the horizontal and vertical modulation factors according to the coordinate indexes. The S-LUT method brings a trivial approximate but achieves less memory usage and faster computational speed at the same time. According to the above statement, the memory usage of a 3D object in S-LUT method depends on the number of the depth planes and will be huge if the number of the depth planes is large. In order to solve this problem, compressed-LUT (C-LUT) method^43^ was developed. In C-LUT method, the horizontal and vertical modulator factors are separated from the depth information. Hence, only the horizontal and vertical modulation factors of the first depth plane need to be pre-calculated and the FPs of different depth planes can be generated by multiplying the corresponding longitudinal modulation factors. C-LUT method is based on an approximation where the depth of the 3D object is considerably smaller than the distance between the object and the hologram. As a result, there is distortion in the reconstructed images and the distortion increases with the increment of the object depth. Later, accurate C-LUT method^44^, accurate high C-LUT method^45^ was proposed in succession to alleviate the distortion by executing additional exponential computation.

Another way to speed up the calculation in point-based method is to reduce the calculation region of each object point. Inspired by this idea, Shimobaba et al. developed wavefront recording plane (WRP)^46^, which is a virtual plane and placed between the 3D object and the hologram, to accelerate the computation. This method consists of two steps. In the first step, the complex amplitude distribution of a 3D object on the WRP is recorded by point-based method. In the second step, the complex amplitude distribution on the CGH is calculated by executing Fresnel diffraction of convolution form between the WRP and CGH. Since WRP has the amplitude and phase information of a 3D object, the diffraction calculation from the WRP to the CGH is equivalent to calculate the complex amplitude distribution on the CGH from a 3D object directly. If the WRP is placed to the 3D object closely and the thickness of the 3D object is small, each object point will emit a wavefront covering only a small region on the WRP due to the restriction of the maximum diffraction angle^47^. In this way, the computation burden can be reduced dramatically because the size of the FPs on the WRP is much smaller than that of the CGH^48^. In order to obtain further acceleration, LUT method can be used in the first step^49,50^. From above, it is obvious that WRP method is very efficient in dealing with the 3D objects within small depth range because the size of the FPs on the WRP is proportional to the distance between the object point and the WRP. In other words, the computation efficiency will decrease dramatically if the depth range of the 3D object is large. In order to address this problem, double WRP^51^ and multiple WRP^52^ methods were proposed. In these methods, the 3D object is partitioned into two or more regions with small depth range along the axial direction. Then WRPs are placed near each region to record the wavefront of the corresponding region and the CGHs are finally generated by summing the contributions of all WRPs. In addition, tilted WRP method was also proposed to generate CGHs of a deep structure object scene^53^. Furthermore, the wavelet transform was also employed to reduce the amount of computation in WRP method^54,55^.

### Polygon-based method

Although numerous fast calculation methods based on point-based method have been proposed, it is still time-consuming due to the huge amount of object points. In order to accelerate the calculation, researchers developed polygon-based method, which regards the 3D object as thousands of polygons rather than millions of points. In this way, the amount of the computational unit is significantly decreased. In polygon-based method, each polygon is regarded as a polygonal aperture, and the CGHs are obtained by adding the diffraction patterns of all polygonal apertures. In addition, combining with the rendering algorithms of computer graphics, polygon-based method can easily add texture and shade to the 3D scene. Usually, a 3D object is divided into thousands of tilted polygons which are not parallel to the hologram plane in polygon-based method. Hence, the core issue in polygon-based method is the diffraction calculation between the tilted plane and the hologram plane. Currently, polygon-based method can be divided into four categories, including traditional polygon-based method, full analytical polygon-based method, spatial approximate polygon-based method, and 3D affine transformation polygon-based method.

In polygon-based method, a 3D object is always investigated in two coordinate systems. One is the local coordinate system; the other is the global coordinate system. In implementation, traditional polygon-based method^56–59^ depicts the tilted polygon in the local coordinate system, computes the spectrum by FFT and the new frequency from the 3D rotational transformation matrix, rotates the spectrum to the global coordinate system by interpolation. After the spectrum propagates to the hologram plane in the global coordinate system, the diffraction pattern can be calculated by inverse FFT. For each polygon, traditional polygon-based method must conduct one polygon depiction, one FFT, and one 2D linear interpolation, which are time-consuming. Nevertheless, traditional polygon-based method can add texture for each independent facet and reconstruct realistic 3D scene.

In order to alleviate the heavy computation of traditional polygon-based method, full analytical polygon-based method was proposed, which aims to establish a connection between the spectrum of the primitive polygon and that of the arbitrary polygon^60,61^. Full analytical polygon-based method defines a primitive polygon and computes the analytical spectrum firstly. Then it implicitly uses the Fourier analysis of the 2D affine transformation, the 3D rotational transformation, and the angular spectrum propagation to compute the diffraction pattern in the hologram plane from the analytical spectrum. Although full analytical polygon-based method avoids polygon depiction and FFT for each polygon, it needs extra diffusion and texture adding computation to reconstruct realistic 3D objects, which are both time-consuming.

Spatial approximate polygon-based method uses the precomputed diffraction pattern of the primitive polygon^62^ to calculate the diffraction pattern of the tilted polygon approximately, which has the translational, rotational, and scaling relation with the primitive polygon. This method avoids FFT because all the computations are in the spatial domain and can add diffusive information without extra computation. However, the reconstruction distance is constrained and the texture cannot be added for each independent polygon.

Traditional 3D affine transformation polygon-based method^63^ defines a primitive polygon with an amplitude and a phase function in the local coordinate system and computes its spectrum firstly. Then the pseudo inversion matrix is used to calculate the core parameters in the 3D affine transformation matrix from the vertex vectors of the primitive polygon and the tilted polygon. Finally, the CGHs can be calculated in one step using the core parameters in the 3D affine transformation matrix because the 3D affine transformation contains the translational, rotational, and scaling transformation in 3D space. This method saves the time in polygon depiction and FFT, needs no extra diffusion computation and has no depth limitation. To speed up the computation, full analytical 3D affine transformation polygon-based method^64,65^ was also proposed, where one primitive triangle is defined and its analytical spectrum is explicitly expressed. The global angular spectrum of an arbitrary polygon in the hologram plane is calculated by using the analytical spectrum of the primitive triangle and 3D affine transformation matrix. Moreover, LUT method was also applied in polygon-based method to speed up the calculation by pre-calculating and storing the analytical spectrum in a table^66^.

In conclusion, polygon-based method has less computational unit compared with point-based method. However, the computation in each unit is more complex, such as depiction and interpolation. Although several improved polygon-based methods have been proposed to avoid above complex computation, they need extra diffusion and texture adding computation, which are also time-consuming.

### Layer-based method

Point-based method and polygon-based method can provide precise geometrical information of a 3D scene, whereas the amount of the computational unit is extremely large. In recent years, layer-based method was developed to reduce the computational unit and accelerate the computation^67^. In layer-based method, the 3D object is divided into several layers parallel to the hologram plane and each layer is regard as an independent computational unit. Then Fresnel diffraction is used to calculate the sub-hologram of each layer and the CGHs are obtained by the superimposition of all sub-holograms. Considering the limited accommodation resolution of human eyes, the computational unit in layer-based method is small compared with point-based method and polygon-based method. Afterwards, the angular-spectrum method was also used in layer-based method to avoid the paraxial approximation and calculate accurate diffracted field^68^. From above, it is obvious that 2D FFT is the basic mathematics tool in layer-based method and limits the calculation speed no matter what kind of diffraction method is used. Accordingly, some improved methods were proposed to speed up the calculation, such as non-uniform 2D FFT method^69^, sub-sparse 2D FFT method^70^ and so on^71,72^.

## Image quality enhancement and speckle suppression

In holographic display, the CGHs are always loaded on the SLM to acquire the target wavefront, so the feature of the SLM has great influence on holographic display effect. At present, most commercial SLMs can hardly modulate amplitude and phase simultaneously and independently. In other words, there is information loss in CGH encoding process, which degrades the reconstruction quality. The reconstructed image is composed of spots and each spot has an area known as the Airy disk. Hence, the actual intensity of the reconstructed image is determined by not only the amplitude distribution but also the phase distribution due to the interference in the overlapping spot areas. It is well known that random phase (RAP) is widely utilized to diffuse the object information in the hologram plane in CGH calculation. As a result, there are unwanted interferences between the adjacent pixels due to the phase differences, which are known as the speckle noise. In the last decades, numerous optimized algorithms have been developed to enhance the reconstruction quality and suppress the speckle noise. From the principle, the algorithms can be divided into phase optimization method, complex amplitude modulation (CAM) method and other methods.

### Phase optimization method

**Fig. 4:**
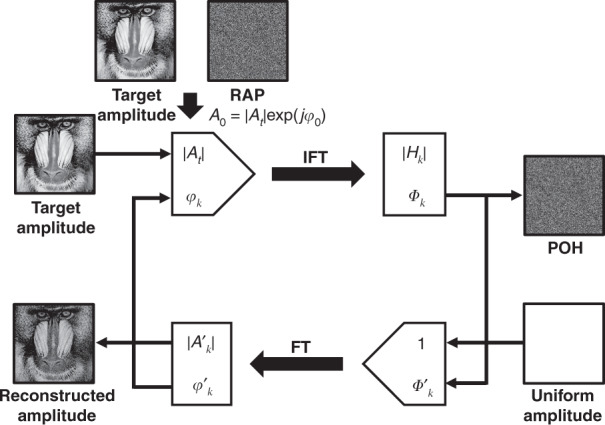
Flowchart of GS algorithm.

The traditional phase optimization method is iterative algorithm. Gerchberg-Saxton (GS) algorithm is a widely used iterative algorithm to optimize the phase distribution^73^ as shown in Fig. 4. To begin with, the RAP *φ*_0_ is used to multiply the target image as the initial input complex amplitude *A*_0_ = | *A*_*t*_ | exp(*jφ*_0_) in the image plane, where |*A*_*t*_ | is the amplitude distribution of the target image. In each iteration, there are four steps as shown in Eq. (3): (1) the complex amplitude field *H*_*k*_ = |*H*_*k*_ | exp(*jΦ*_*k*_) in the hologram plane is calculated by inverse Fourier transform (IFT) of the complex amplitude field *A*_*k*_ = |*A*_*t*_ | exp(*jφ*_*k*_) in the image plane, where *k* represents the *k*th iteration; (2) the phase-only hologram (POH) *H'*_*k*_ = exp(*jΦ'*_*k*_) is generated from the complex amplitude field *H*_*k*_ after phase extraction and amplitude normalization; (3) the reconstructed complex amplitude field *A'*_*k*_ = |*A'*_*k*_ | exp(*jφ'*_*k*_) in the image plane is calculated by Fourier transform (FT) of the POH *H'*_*k*_; (4) the input complex amplitude *A*_*k+*1_ for the next iteration is generated after the reconstructed amplitude |*A'*_*k*_ | is replaced by the target amplitude |*A*_*t*_ | . In implementation, GS algorithm reduces the errors within the first few iterations rapidly, whereas the convergence slows down or even stagnates in subsequent iterations.

3 $$\begin{array}{lll}H_k &=& IFT(A_k)\\ H^{\prime}_k &=& \frac{{H_k}}{{|H_k|}}\\ A^{\prime}_k &=& FT(H^{\prime}_k)\\ A_{k + 1} &= &\left| {A_t} \right|\frac{{A^{\prime}_k}}{{\left| {A^{\prime}_k} \right|}}\end{array}$$

In order to speed up convergence, Fienup algorithm was developed. The first three steps of Fienup algorithm are the same as GS algorithm. In the fourth step, the linear combination of the target amplitude and the reconstructed amplitude is used as the input amplitude for the next iteration instead of imposing the target amplitude directly^74^ as shown in Eq. (4). Afterwards, the nonlinear combination of the target amplitude and the reconstructed amplitude was also presented to improve the computation efficiency^75^.

4 $$\begin{array}{c}A_{k + 1} = \left| {A_{k + 1}} \right|\frac{{A^{\prime}_k}}{{\left| {A^{\prime}_k} \right|}}\\ \left| {A_{k + 1}} \right| = \left| {A_t} \right| + \alpha (\left| {A_t} \right| - \left| {A^{\prime}_k} \right|)\end{array}$$

where *α* is set for enhancing the convergence.

Although GS algorithm and Fienup algorithm can achieve effective convergence, they always stagnate into the local minimum. Instead of imposing amplitude constraint in the entire image plane, Fidoc algorithm has introduced amplitude freedom in the image plane to improve the reconstruction quality^76^. In Fidoc algorithm, the image plane is partitioned into signal region and freedom region. In each iteration, the amplitude distribution in the signal region is constrained, whereas the constraint in the freedom region is relaxed as shown in Eq. (5). Along this way, the noise suppression parameter^77^ and weighted constraint iterative algorithm^78^ were developed in succession to suppress the noise in the freedom region and improve the diffraction efficiency.

5 $$\left| {A_{k + 1}} \right| = M[\left| {A_t} \right| + \alpha (\left| {A_t} \right| - \left| {A^{\prime}_k} \right|)] + \gamma (1 - M)\left| {A^{\prime}_k} \right|$$

where *α* is set for enhancing the convergence, *γ* is set for suppressing the noise and *M* is a 2D matrix, the value in the signal region is 1 and the value in the freedom region is 0.

Iterative algorithms can optimize the phase distribution effectively by repeating forward and backward diffraction calculation between the image plane and the hologram plane. However, they are time-consuming and not suitable to realize dynamic display. Hence, some non-iterative algorithms have also been proposed to improve the reconstruction quality. As we know, the initial RAP with 2π range causes excessive diffusion of the object information, especially for high frequency region. In other words, it does not need RAP with 2π range as the initial phase to be added on the target image. Inspired by this idea, limited random phase method^79^, where RAP with 1.2π range is provided for the gray image and RAP with 1.5π range is added on the binary image, and gradient-limited random phase^80,81^, where RAP with different ranges is provided for the low frequency region and the high frequency region of the target image, were proposed successively. Furthermore, a frequency-based optimized random phase method^82^ was also developed, where RAP with different frequency ranges and maximal values is added on different frequency regions of the target image with the help of the Butterworth filte. Although above methods can enhance the reconstruction quality, the speckle noise could not be suppressed completely due to the presence of the RAP. Recently, patterned-POH method was proposed, where a tiled random phase mask is added to the desired image^83^. The tiled random phase mask acts as a diffuser and smooths the magnitude of the diffracted waves to a near uniform distribution. In addition, several RAP-free methods were also proposed to suppress the speckle noise. In RAP-free methods, virtual convergence light^84,85^ and quadratic phase^86,87^ with continuous distributed spectrum were utilized as the initial phase distribution of the desired image to generate Fresnel and Fourier POHs, respectively. Owing to the avoidance of the RAP, RAP-free methods can suppress the speckle noise effectively.

**Fig. 5:**
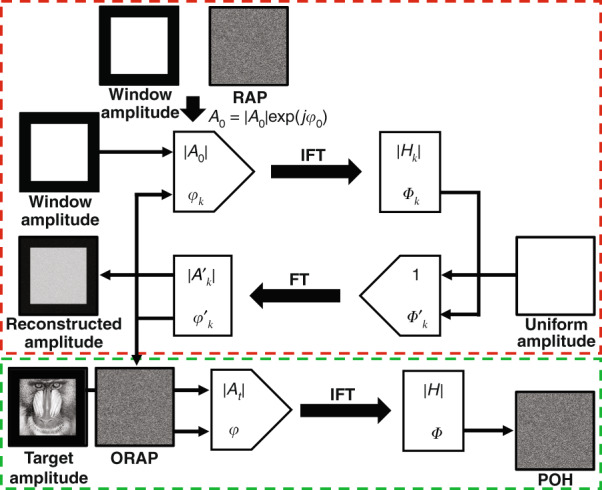
Flowchart of ORAP method.

Moreover, optimized random phase (ORAP) method^88^, which combined iterative algorithms and non-iterative algorithms, was also developed to implement the generation of POHs for arbitrary target images. This method consists of off-line calculation and on-line calculation as shown in Fig. 5, where the red dashed line represents off-line calculation and the green dashed line represents on-line calculation. In off-line calculation, a target window |*A*_0_ | corresponding to the size of the target image is multiplied by the random phase *φ*_0_ as the initial input complex amplitude *A*_0_ = | *A*_0_ | exp(*jφ*_0_) in the image plane and GS algorithm is performed to optimize the phase of the reconstructed target window. Once the GS algorithm is completed, the phase of the reconstructed target window *φ'*_*k*_ (ORAP) can be taken as the initial phase distribution *φ* of arbitrary target images |*A*_*t*_ | with the same size of the target window to generate POHs *Φ* as shown in Eq. (6). ORAP method avoids the on-line iterative calculation and is suitable for dynamic holographic display. Thereafter, ORAP method was extended to Fresnel region with the help of iterative Fresnel transform algorithm^89^. Moreover, tiled random phase mask^90^ and quadratic phase^91^ were also combined with ORAP method to suppress the speckle noise and achieve high-quality reconstruction.

6 $$\Phi = {\rm{angle}}\{ {\rm{IFT}}(|{\rm{A}}_{\rm{t}}|\exp ({\rm{j}}\varphi ))\}$$

where *angle* represents phase extraction operation.

### CAM method

**Fig. 6:**
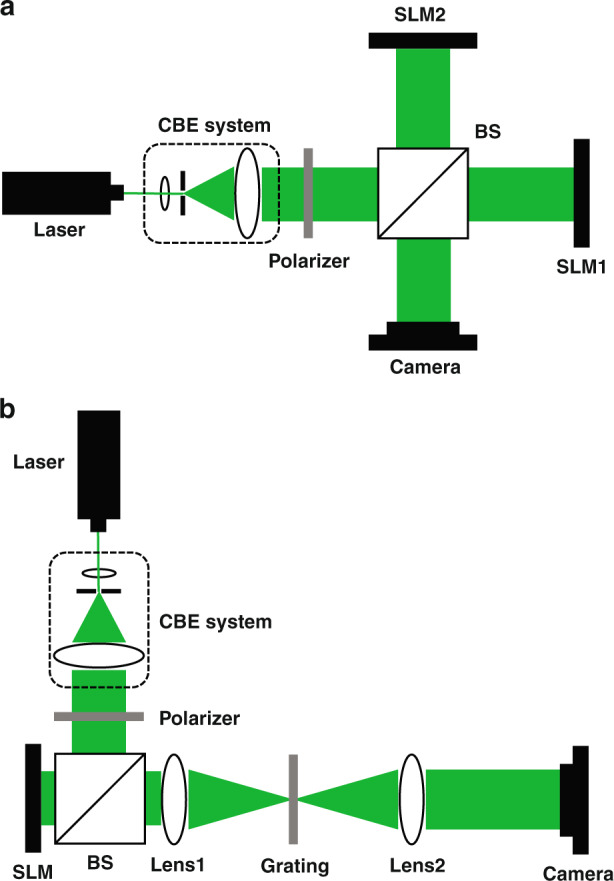
Optical system of device method based on CAM. **a** Interference method; **b** grating method, where CBE system is collimating and beam expanding system.

As mentioned above, there is information loss when the CGHs are loaded on the SLM. To avoid the information loss and realize CAM, both device method and encoding method have been proposed. So far, device method can be mainly classified into two categories according to the number of SLMs in the optical system. One is device method based on multiple SLMs; the other is device method based on single SLM. In 1984, Bartelt used two phase-only transmissive elements as spatial filters and auxiliary optical system to form a complex amplitude spatial filter^92^. Subsequently, several cascaded methods for different types of liquid crystal displays were proposed and demonstrated^93–96^. Afterwards, inspired by the superposition of light waves in the interferometer, researchers proposed interference method^97,98^ to achieve CAM, where the complex hologram is decomposed into two amplitude-only holograms (AOHs) or POHs based on analytic formula as shown in Eq. (7) and the decomposed two holograms are uploaded on two SLMs which are placed in the interferometer as shown in Fig. 6a. Thereafter, iteration and phase compensation method^99^ was also proposed. In this method, the POH uploaded on the first SLM is calculated by iterative algorithm according to the target amplitude and the second SLM is used to compensate the phase reconstructed by the first SLM. Moreover, a system which can control the amplitude and phase arbitrarily by changing the phase distribution loaded on two polarization-dependent SLMs with the assistance of a polarizer and a half-wave plate^100^ was also designed. As mentioned above, it is obvious that the optical structure of device method based on multiple SLMs is complicated and the alignment is hard to be achieved no matter what kind of system is used.

7 $$\begin{array}{c}A\exp (j\varphi ) = A\cos \varphi + jA\sin \varphi \\ A\exp (j\varphi ) = \exp (j\theta _1) + \exp (j\theta _2)\end{array}$$

where *A*exp(*jφ*) is the complex hologram, *A*cos*φ* and *A*sin*φ* are the decomposed two AOHs, exp(*jθ*_1_) = exp{*j*[*φ* + cos^−1^(*A*/2)]} and exp(*jθ*_2_) = exp{*j*[*φ* − cos^−1^(*A*/2)]} are the decomposed two POHs.

In order to simplify the optical system and avoid alignment, several device methods based on single SLM were designed to achieve CAM in the last decade. In 2011, Liu et al. uploaded the decomposed two AOHs in different regions of the SLM and utilized an amplitude grating filter as a frequency filter to couple them to a complex hologram in the output plane of the 4 f system^101^. Afterwards, the combination of two POHs and an amplitude grating or a phase grating^102,103^ was also utilized to generate a complex hologram as shown in Fig. 6b. Besides, grating filter can also synthesize two POHs arranged in a checkerboard pattern to a complex hologram with the help of a microprism array^104^. Although grating filter is widely used to generate the complex hologram, it introduces extra orders and reduces the efficiency for luminous energy utilization. In addition to grating filter, the combination of a structured half-wave plate, a birefringent crystal and a polarizer^105^ was used to control the amplitude and phase accurately and independently. However, the polarizer also reduces the efficiency for luminous energy utilization. Moreover, the iteration and phase compensation method was optimized by using a single SLM with the assistance of a reflective concave mirror^106^. Although this scheme simplifies the optical system, the iterative calculation is still time-consuming.

**Fig. 7:**
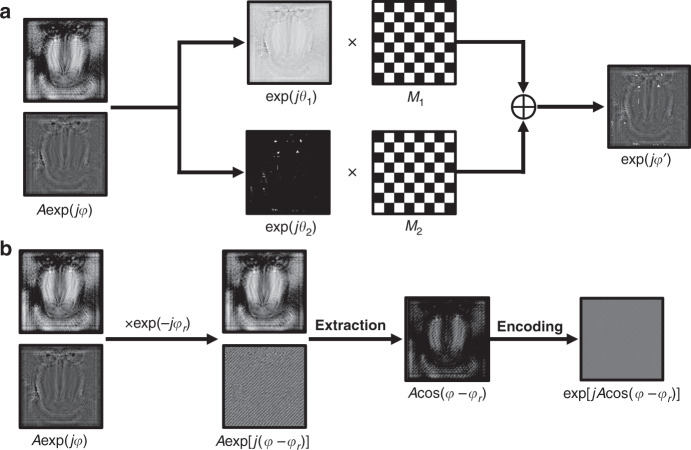
Flowchart of encoding methods based on CAM. **a** DPH method based on single-pixel modulation; **b** hologram bleaching method.

Compared with device method, encoding method is more convenient and has been widely applied in holographic display field. A well-known encoding method to generate complex amplitude field is superpixel method^107–110^. A superpixel contains several actual pixels of the SLM and represents a pixel of the target complex amplitude. In implementation, the actual value loaded on each pixel of the SLM is a part of the target complex amplitude which meets the modulation characteristics of the SLM. According to the sampling theorem, the light wave is resampled after passing through a low-pass filter and the CAM can be achieved successfully by controlling the aperture size of the filter. Working along this direction, double-phase hologram (DPH) method based on single-pixel modulation^111–113^ was also proposed to achieve compact arrangement of each decomposed part and high utilization efficiency of the spatial bandwidth product (SBP). In this method, the target complex amplitude is decomposed into two POHs and complementary 2D binary gratings (checkerboard patterns) are utilized to sample the POHs and combine them into a DPH as shown in Fig. 7a and Eq. (8). Compared with superpixel method, this method ensures that the number of sampling points between the target image and the hologram is always consistent and has high computational efficiency. However, due to the complementary sampling, the reconstructed results are affected by the approximation of the adjacent pixels and the reconstruction accuracy is slightly lower than that of superpixel method. No matter superpixel method or single-pixel method, the spatial shifting noise cannot be eliminated completely by filtering system due to the distribution characteristics of the envelope function. Hence, the weight factor^114^ and band-limiting function^115^ were proposed successively to suppress the spatial shifting noise.

8 $$\exp (j \varphi^{\prime} ) = \exp (j \theta _1)M_{1} + \exp (j \theta _{2})M_{2}$$

where exp(*jθ*_1_) = exp{*j*[*φ* + cos^−1^(*A*/2)]} and exp(*jθ*_2_) = exp{*j*[*φ* − cos^−1^(*A*/2)]} are decomposed two POHs, *Aexp(jφ)* is the target complex amplitude, *M*_*1*_ and *M*_*2*_ are complementary 2D binary gratings (checkerboard patterns), and *exp(iφ')* is the DPH.

Another kind of encoding method for CAM is hologram bleaching method^116–118^. In this method, the amplitude information is encoded as a part of the phase information after the target complex amplitude interferes with an off-axis reference plane wave as shown in Fig. 7b. According to the property of Bessel function, the −1 order of the expansion is the desired complex amplitude as shown in Eq. (9), which can be picked up by a band-pass filter in reconstruction. Working along this direction, the band-limited zone plates and intensity modulation coefficient were introduced to modulate the bandwidth and intensity of the target complex amplitude^119^.

9 $$\begin{array}{lll}H &= &\exp [jA\cos (\varphi - \varphi _r)] \\&= &\mathop {\sum}\limits_{m = - \infty }^\infty {J_m(A)j^m} \exp [ - jm(\varphi - \varphi _r)]\\ H_{ - 1} &= & J_{ - 1}(A)j^{ - 1}\exp [j(\varphi - \varphi _r)]\\ &\approx & jA\exp [j(\varphi - \varphi _r)]/2\end{array}$$

where *H* is the encoded hologram, *H*_-1_ is the −1 order of the expansion, *J*_*m*_(*) is the *m* order of the Bessel function of the first kind. *Aexp(jφ)* is the target complex amplitude, *exp(-jφ*_*r*_*)* is the off-axis reference plane wave.

In addition to the above two encoding methods, double-constraint iterative method was also introduced to modulate amplitude and phase simultaneously. Compared with Fidoc algorithm, the amplitude and phase in the signal domain are both constrained and replaced by the desired values^120,121^ as shown in Eq. (10). In this way, the optimized POHs can reconstruct the desired complex amplitude distribution approximatively. The optical system of double-constraint iterative method is simple. However, iterative calculation is time-consuming and can only generate an approximate result rather than an accurate one.

10 $$A_{k + 1} = M(2\alpha \left| {A_t} \right| - \beta \left| {A^{\prime}_k} \right|) \cdot {{{\mathrm{exp(}}}}j\varphi _t{{{\mathrm{)}}}} + \gamma (1 - M)A^{\prime}_k$$

where *M* is a 2D matrix, the value in the signal region is 1 and the value in the freedom region is 0. |*A*_*t*_ | is the desired amplitude distribution, *φ*_*t*_ is the desired phase distribution, which is usually chosen as uniform phase or quadratic phase. *α, β* and *γ* are set for enhancing the intensity and contrast in the signal domain.

### Other methods

In addition to phase optimization method and CAM method, there are also some other methods for speckle noise suppression. As mentioned above, the speckle noise exists due to the unwanted interferences between the adjacent object points caused by RAP in reconstruction process. Because of the independence and randomness of the RAP, the unwanted interferences are independent and random. Inspired by this idea, researchers proposed time-average method^122–126^ to suppress the speckle noise by summating multiple images generated by different RAP. Although this method is successful in reducing the speckle noise, it requires a SLM with high frame rate and is difficult to realize dynamic holographic display.

Another solution for speckle noise suppression is to avoid the interferences between the adjacent object points. Based on this idea, pixel separation method^127–129^ was proposed, where the CGHs of the sparse images are generated at different time and combined to form the complete image. However, the combination of multiple sparse images is also time-consuming and not suitable for dynamic holographic display. Afterwards, a sampled POH method^130^, which down-samples the desired image with a uniform grid-cross lattice prior to the generation of the POH, was also presented to preserve good visual quality on the reconstructed image. Working along this direction, the edge dependent down-sampled lattice^131^, the complementary down-sampled lattice^132^ and an optimal sampled phase-only hologram method^133^ were presented sequentially to improve the reconstruction quality. Although the above methods can suppress the speckle noise effectively, the down-sampling causes the loss of information.

Moreover, partially coherent light (PCL) illumination is also a feasible way to suppress the speckle noise by reducing the coherence of the light source^134,135^. In the last decade, many scholars have studied the influence of PCL illumination on holographic reconstruction^136–138^ and designed various CGH algorithms^139,140^. However, PCL illumination will result to the problem of image blurring, which is fatal for display. Hence, coherent light is still the main illumination source for holographic display due to the high brightness and contrast.

## Color display

**Fig. 8:**
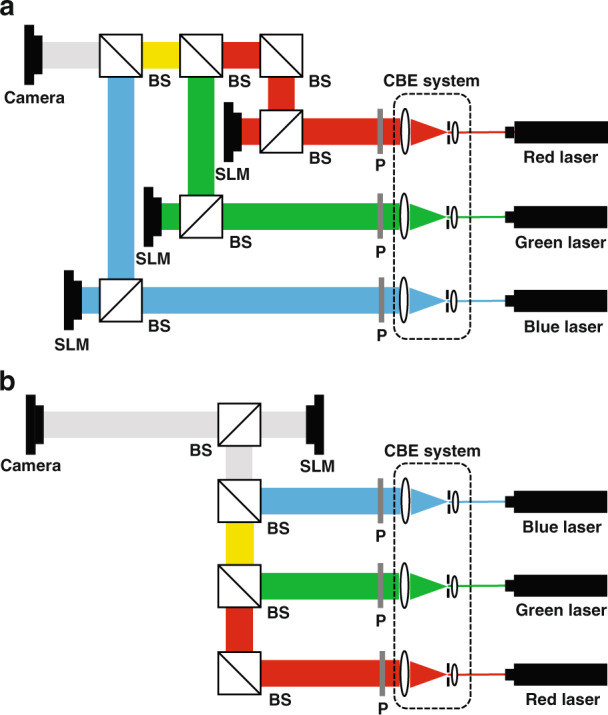
Optical system of color holographic display. **a** Spatial-multiplexing method; **b** time-multiplexing method, where P is polarizer and CBE system is collimating and beam expanding system.

Since holography is based on interference and diffraction theory, red (R), green (G), blue (B) holograms of the color object need to be generated and reconstructed separately. Currently, the most widely used method to realize color holographic display is spatial-multiplexing method as shown in Fig. 8a, where three SLMs are required to load RGB hologram, respectively^141–143^. In reconstruction, RGB lasers illuminate on the corresponding SLMs and the color object is formed by combining RGB objects using BSs. The system based on spatial-multiplexing method fully utilizes the SBP of the SLM and has high optical efficiency. However, the display system is too bulky in size and too high in cost because the optical elements used in spatial-multiplexing method trebles that of monochromatic holographic display. Moreover, the system has the problem of precise alignment for RGB reconstructed objects. To reduce the number of SLMs required in color holographic display, time-multiplexing method was proposed^144–146^ as shown in Fig. 8b. In time-multiplexing method, RGB lasers illuminate on a single SLM time-sequentially and the corresponding holograms are loaded on the SLM synchronously. When the frequency of switching RGB lasers and the corresponding holograms is high enough, the color objects can be observed by the persistence effect of human eyes. This system requires a SLM with very high frame rate. Besides, it also needs the accurate synchronization of the RGB laser illumination and the corresponding hologram loading. In addition, an improved spatial-multiplexing method^147,148^ was proposed to reduce the system size, where one SLM is divided into three regions for RGB holograms and illuminated by spatially separated RGB lasers. However, it is obvious that the decrease of effective resolution for RGB holograms deteriorates the reconstruction quality.

**Fig. 9:**
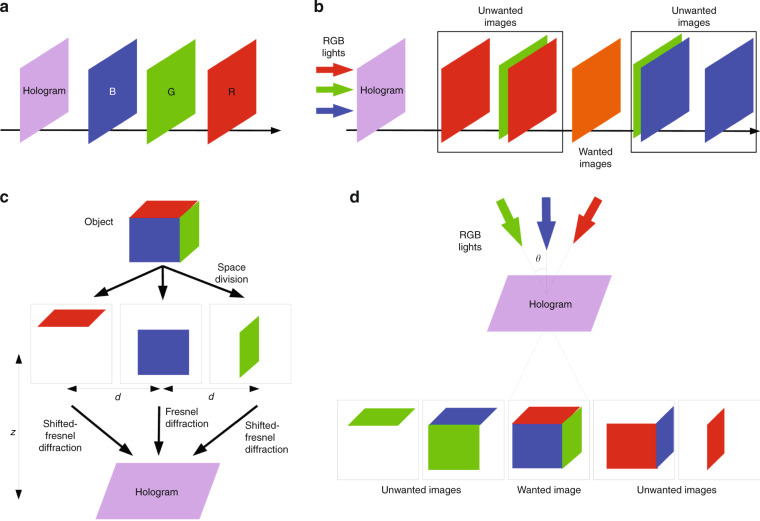
Recording and reconstruction processes of DDM and SDM. **a** Recording and **b** reconstruction processes of DDM; **c** Recording and **d** reconstruction processes of SDM, where *z* is the diffraction distance, *d* is the distance between each transverse component and *θ* is the incident angle of the reconstruction light. Reprinted with permission from ref. ^151^. ©The Optical Society.

In the last decade, various CMC methods have been developed as an alternative to spatial-multiplexing method and time-multiplexing method to realize color holographic display. In these methods, RGB holograms are synthesized into a color-multiplexing hologram (CMH) by multiplexing different physical parameters. In 2008, depth-division multiplexing (DDM) method^149,150^ was developed to reconstruct color 2D images as shown in Fig. 9a, b. In DDM method, the distance from the CMH to RGB images is predetermined and an iterative multi-plane optimization algorithm is applied to improve the final reconstruction quality. When the CMH is illuminated by RGB lasers simultaneously, the color image can be reconstructed at the predetermined position. DDM achieves color holographic display by a single SLM successfully. However, it can only reconstruct color 2D images rather than 3D objects and is difficult to realize dynamic display because of the iterative computation. Afterwards, space-division multiplexing (SDM) method^151^ was presented to reconstruct color 3D objects as shown in Fig. 9c, d. SDM divides the color 3D object into transversely distributed RGB components and utilizes Fresnel diffraction or Shifted-Fresnel diffraction to calculate RGB holograms, respectively. In reconstruction, the color 3D object can be obtained by illuminating RGB lasers to the CMH at the predetermined angle simultaneously. Due to the transverse distribution of RGB components, the unwanted images are separated from the desired object in space domain. It is noted that RGB holograms are optimized individually by GS algorithm for improvement of the reconstruction quality in SDM method, which indicates that it is also not suitable for dynamic holographic display.

**Fig. 10:**
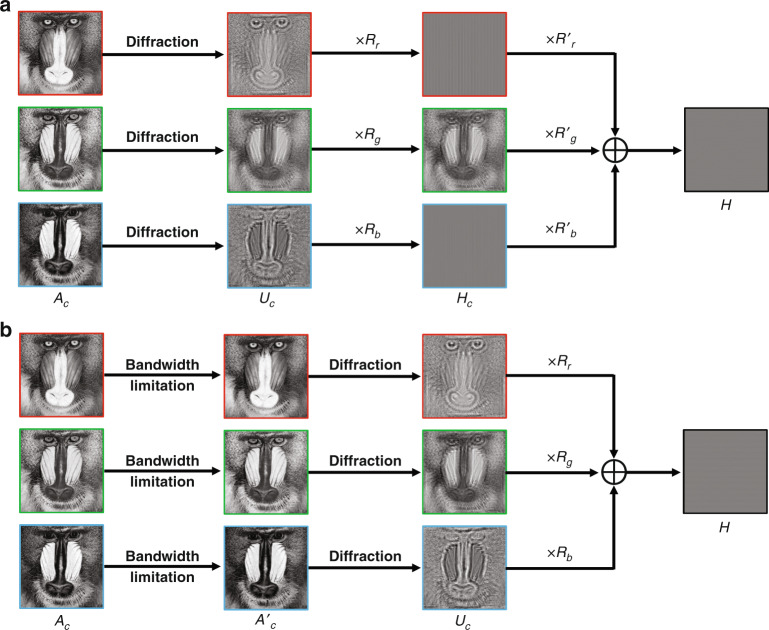
Flowchart of CMC methods. **a** The method in ref. ^152^, where *c* = *r, g, b* corresponds to RGB channel, *A*_*c*_ is the target image, *U*_*c*_ is the diffraction pattern of *A*_*c*_, *R*_*c*_ = *exp(jk*_*c*_*xsinθ*_*c*_*)* is the tilted plane reference light along *x* axis, *H*_*c*_ = *U*_*c*_ *×* *R*_*c*_ is the off-axis hologram, *R'*_*c*_ = *exp(jk*_*c*_*ysinθ)* is the titled plane reference light along *y* axis, and *H* is the complex CMH; **b** The method in ref. ^157^, where *A'*_*c*_ is the target image after bandwidth limitation, *U*_*c*_ is the diffraction pattern of *A'*_*c*_, *R*_*c*_ = *exp(jk*_*c*_*ysinθ)* is the titled plane reference light along *y* axis, and *H* is the complex rainbow hologram.

In 2014, another multiplexing method^152^ was also proposed to achieve color holographic display by using off-axis CMHs as shown in Fig. 10a. In this method, RGB diffraction patterns interfere with the corresponding tilted plane reference lights along *x* axis to generate RGB off-axis holograms. Then RGB off-axis holograms are synthesized and encoded as a phase-only CMH based on analytic formula after interfering with titled plane reference lights along *y* axis. In reconstruction, RGB lasers are separated to accommodate the illumination angle of off-axis RGB holograms and the desired images can be picked up by a band-pass filter in the frequency plane. This method can be easily applied for color dynamic holographic display because there is no iterative calculation. However, the system is complex and unstable due to the arrangement of the illumination source. Subsequently, a specially designed optical element, which consists of two prisms, a transparent glass and RGB color filters, was used to filter and refract the white light source into RGB colors with different directions^153^. The designed optical element greatly simplifies the complexity of the system based on off-axis CMH.

In recent years, several frequency-division methods (FDMs) were successively developed to realize color holographic display^154–156^. FDM divides the frequency spectrum of RGB components at distinct location in the Fourier plane of a CMH. In this way, the color object can be reconstructed successfully with the assistant of a filter in the frequency plane when a multi-wavelength light source is used as the illumination source. FDM simplifies the display system because of the on-line illumination. Another holographic display technology with white light illumination is rainbow holography. Recently, a rainbow hologram alike method^157,158^ based on frequency domain multiplexing has also been proposed for color holographic display as shown in Fig. 10b. In implementation, RGB diffraction patterns are calculated after the bandwidth of RGB components is limited by a slit filter in the vertical direction. Then RGB diffraction patterns are synthesized as a rainbow hologram after interfering with the plane reference lights with an off-axis angle *θ* along *y* axis. In reconstruction, the rainbow hologram is illuminated by white light with an off-axis angle *-θ* along *y* axis and a slit filter is used to extract the desired RGB frequency components to achieve color display. It is noted that the color of the reconstructed object can be changed by changing the position of the slit filter as well as the width of the slit filter Fig. 10.

## Unwanted terms and modulation accuracy

### Unwanted terms

In holographic display, there is always a zero-order beam in the center of the reconstructed image due to the pixelated structure of the SLM^159^. Specifically, the incident light is are modulated by not only the active areas but also the inactive areas between the adjacent pixels^160^ and the zero-order beam is determined by the fill factor, the amplitude and the phase of the inactive areas^161^. In order to eliminate the zero-order beam, two kinds of methods have been proposed. One is to translate the reconstructed image from the zero-order beam by superimposing a linear phase and a divergent spherical phase^161^, or an additional phase checkerboard function^162^ to the CGHs, the other is to produce a cancellation beam which destructively interferes with the zero-order beam^163–165^. Apart from the zero-order beam, the conjugate image always appears in the AOH due to the coding process from the complex hologram to the nonnegative AOH. Off-axis recording could separate the conjugate image from the desired image in the spatial frequency domain by adding a carrier frequency on the CGHs. In addition, single-sideband method can also eliminate the conjugate image by filtering out half of the spatial frequency in both recording and reconstruction process^166,167^. Because of the pixelated structure of the SLM, the diffraction pattern is always replicated laterally, which is known as the high-order diffraction terms. In order to eliminate the high-order diffraction terms, a 4 f system with a filter in the frequency plane can be used in optical experiments^168^. Moreover, the diffraction pattern is modulated by an envelope function caused by the finite pixel size. In order to improve the visual effect, digital pre-filtering method was presented to compensate the distortion^169^.

### Modulation accuracy

Although the SLM can modulate the amplitude or phase of the incident light, the accurate wavefront manipulation is still hindered by some errors. In other words, in order to achieve high-precision holographic display, the modulation errors caused by the SLM cannot be ignored. Generally, the modulation accuracy of the SLM is mainly influenced by two aspects: the static error and the dynamic error. The static error is introduced by the flatness of the cover glass, while the dynamic error is caused by the nonuniform and nonlinear gamma curve. In practical applications, a compensation matrix for the panel of the SLM or a remapping LUT for the gamma curve is built after the modulation capability of the SLM is measured and then the calibration information is added to the CGH to achieve precise encoding. Until now, several approaches have been proposed to compensate the static error. Xun et al. utilized the four-step phase-shift interference algorithm to measure and compensate the nonuniform flatness of the backplane curvature^170^. Otón et al. developed a multipoint calibration method to improve the compensation performance and calibrated the error by Michelson interference and Ronchi grating method^171^. To improve the modulation accuracy, researchers have also made several attempts to overcome the influence from the dynamic error. Reichelt introduced a spatially resolved phase response method to measure and compensate the spatial nonuniformity^172^. Yang et al. employed digital holographic microscopy to characterize the nonlinear dynamic phase response of the SLM and utilized gamma curve calibration to optimize the distortion^173^. Zhao et al. divided the entire panel of the SLM into several local regions and proposed a multi-region calibration method to minimize the nonlinear response and static distortion of each local region^174^. Apart from manual calibration, some automatic calibration software has also been developed for the fast measurement and the automatic compensation. In short, the calibration method has gradually become more precise and faster.

## Conclusion

In this paper, we review numerous CGH algorithms with the aim of color dynamic holographic 3D display. For computation speed, we report various fast calculation algorithms based on point-based method, polygon-based method, and layer-based method. Although many effective algorithms have been developed, they are still isolated and not fast enough. The combination of different primitive methods into an optimized algorithm is a good solution to accelerate CGH computation. In addition, with the development of computer, the combination of fast calculation algorithms and high-performance computing equipment is also an effective means to speed up the calculation^175,176^. Furthermore, deep learning has also been used to CGH generation in recent years^177–180^, which shows great potential for both high-speed and high-quality reconstruction. For image quality enhancement and speckle suppression, we report phase optimization method, CAM method and some other methods. Traditional iterative phase optimization method is time-consuming, whereas non-iterative phase optimization method will cause other problems, such as ringing artifacts or a periodic patterning effect. In recent years, the appearance of ORAP, which combines iterative and non-iterative phase optimization methods, opens a door to realize high-quality dynamic holographic display by optimizing phase distribution. CAM is realized with the reduction of effective resolution because the amount of information of a complex hologram doubles that of an AOH or a POH. In other words, the essence of CAM, no matter how to realize, is sacrificing the SBP to achieve the expression of the complex amplitude information. In order to remit the limitation of finite SBP, some approaches have been proposed^181–184^. At the same time, the development of new optical materials, such as metamaterials, lights up a passage for complex amplitude modulators^185,186^. Temporal method, pixel separation method, and PCL illumination can suppress the speckle noise efficiently, whereas, there is lack of further optimization direction. For color display, we review various CMC methods which aim to realize color holographic display. However, limited by the SBP of existing SLMs, the display effect cannot meet the requirements of humans and needs to be improved further. Despite the CMC method is facing the restriction of finite SBP, it is meaningful to introduce it into color holographic display because it avoids not only the complex structure but also the high cost. With the development of SLMs towards higher resolution, smaller pixel size and more accurate modulation, it is believed that color dynamic holographic 3D display will make a breakthrough in the future^187^. In summary, holographic display is regarded as one of the most promising 3D display technologies because it can reconstruct all the depth cues of a 3D scene. Meanwhile, there is still a great potential for the further development of color dynamic holographic 3D display. With the development of not only the CGH algorithms but also the devices and systems^188,189^, it is expected that holographic display will come into the market and daily life in a near future.
